# *Sporothrix brasiliensis* Infection Modulates Antimicrobial Peptides and Stress Management Gene Expression in the Invertebrate Biomodel *Galleria mellonella*

**DOI:** 10.3390/jof9111053

**Published:** 2023-10-27

**Authors:** Nathália Faria Reis, Myrela Conceição Santos de Jesus, Lais Cavalcanti dos Santos Velasco de Souza, Lucas Martins Alcântara, Julia Andrade de Castro Rodrigues, Simone Cristina Pereira Brito, Patrícia de Azambuja Penna, Cecília Stahl Vieira, José Rodrigo Santos Silva, Bruno de Araújo Penna, Ricardo Luiz Dantas Machado, Hector M. Mora-Montes, Andréa Regina de Souza Baptista

**Affiliations:** 1 Center for Microorganisms’ Investigation, Department of Microbiology and Parasitology, Biomedical Institute, Federal Fluminense University, Niterói 24020-141, RJ, Brazil; nathaliafariareis@id.uff.br (N.F.R.); myrelaj@id.uff.br (M.C.S.d.J.); laiscavalcanti@id.uff.br (L.C.d.S.V.d.S.); martins_lucas@id.uff.br (L.M.A.); julia_andrade@id.uff.br (J.A.d.C.R.); scpbrito@id.uff.br (S.C.P.B.); ricardomachado@id.uff.br (R.L.D.M.); 2 Laboratory of Insect Biology, Federal Fluminense University, Niterói 24220-900, RJ, Brazil; patriciaazambuja@id.uff.br (P.d.A.P.); ceciliastahl@gmail.com (C.S.V.); 3 Department of Statistics and Actuarial Sciences, Federal University of Sergipe, São Cristóvão 49107-230, SE, Brazil; rodrigo.ufs@gmail.com; 4 Laboratory of Gram-Positive Cocci, Biomedical Institute, Fluminense Federal University, Niterói 24020-141, RJ, Brazil; bpenna@id.uff.br; 5 Department of Biology, Division of Natural and Exact Sciences, Campus Guanajuato, University of Guanajuato, Guanajuato 36050, Mexico; hmora@ugto.mx; 6 Rede Micologia RJ—Fundação de Amparo à Pesquisa do Estado do Rio de Janeiro (FAPERJ), Rio de Janeiro 21941-630, RJ, Brazil

**Keywords:** sporotrichosis, gene expression, invertebrates, antimicrobial peptides, zoonosis, immune response

## Abstract

*Sporothrix brasiliensis* is the most pathogenic species, responsible for the Brazilian cat-transmitted sporotrichosis hyperendemic. In this scenario, an investigation of the pathogen–host interaction can provide relevant information for future treatment strategies. To this end, the invertebrate *Galleria mellonella* has proven to be a suitable alternative for evaluating the virulence of pathogenic fungi, since the insect immune system is similar to the mammalian innate immune response. The aim of this work was to investigate phenotypic and molecular aspects of the immune response of *G. mellonella* throughout the *S. brasiliensis* infection. Hemocyte density and the evolution of the fungal load were evaluated. In parallel, RT-qPCR expression analysis of genes encoding antimicrobial peptides (*Gallerimycin* and *Galiomycin*) and stress management genes (*C7 Contig 15362* and *C8 Contig 19101*) was conducted. The fungal load and hemocyte densities increased simultaneously and proportionally to the deleterious morphological events and larvae mortality. *Gallerimycin*, *C7 Contig 15362* and *C8 Contig 19101* genes were positively regulated *(p* < 0.05) at distinct moments of *S. brasiliensis* infection, characterizing a time-dependent and alternately modulated profile. *Galiomycin* gene expression remained unchanged. Our results contribute to the future proposal of potential alternative pathways for treating and consequently controlling *S. brasiliensis* zoonosis, a major public health issue in Latin America.

## 1. Introduction

Sporotrichosis is a disease of great importance to public health, since human infection can occur through direct contact with contaminated soil organic matter (sapronosis) or, more frequently, by the zoonotic route through the domestic feline [1,2]. In the latter scenario, the incriminated fungus is *Sporothrix brasiliensis*, the most virulent and pathogenic species of the *Sporothrix* genus, with strong evidence of drug resistance [3,4]. The *S. brasiliensis*-zoonotic-transmitted sporotrichosis began as an outbreak in the late 1990s in Rio de Janeiro, Brazil, and it is currently considered to be hyperendemic nationwide [5,6], with reports of recent geographical expansion to other Latin American countries [7,8]. Recently, the first three cases of domestic-feline-transmitted *S. brasiliensis* outside South America were described in the United Kingdom, Europe [9].

Itraconazole is the drug of choice for human and feline sporotrichosis [10,11]. However, cat treatment remains a challenge for the veterinarian, given the limited number of antifungal agents, their high cost and adverse effects, and their commercial presentations. Moreover, little is known about the immune response triggered by the infected hosts, especially the domestic feline [1,11].

In order to address this issue, the expansion of studies aiming to expand information related to the pathogen–host interaction is necessary. To this end, *Galleria mellonella* stands out as an invertebrate model for the investigation of the immune response triggered by distinct fungal pathogens, such as yeast of the *Candida* genus [12,13] and *Cryptococcus neoformans* [14]. Among filamentous fungi, *Aspergillus niger*, *Rhizopus* spp. and *Rhizomucor* spp. have also been challenged against the invertebrate biomodel’s defense [15,16,17], as have also the dimorphic *Paracoccidioides lutzii* and *Histoplasma capsulatum* [18,19]. However, few studies have been dedicated to the interaction between *G. mellonella* and fungi of the *Sporothrix* genus, all focusing on phenotypic aspects investigating the host–pathogen interaction [20,21,22] or in vivo drug response [23].

The *G. mellonella* immune system has close structural and functional similarities to the mammalian innate immune system [24,25], and it is divided into two strongly interconnected pathways: the cell-mediated pathway and the humoral pathway [26]. The cellular response of insects is mediated by hemocytes—the phagocytic cells in the hemolymph. These have the function of phagocytosing foreign bodies, capturing and encapsulating them in multicellular structures called nodules or capsules [26,27,28], playing roles analogous to those of human macrophages and neutrophils [24,25]. In parallel, the humoral response of insects includes antimicrobial peptides (AMPs), which act directly against invaders. Among the *G. mellonella* AMPs, gallerimycin and galiomycin are the most well-described in the literature [29], with evidence of positive regulation on the encoding genes’ expression in fungal infections with this insect, such as *Candida* spp. and *Aspergillus niger* [17,30,31].

The transcriptome analysis developed for *G. mellonella* provided important data related to immunity, notably expanding the knowledge about immune and stress-inducible genes in the invertebrate model. These results provided access to the genetic architecture of the insect’s immunity and elucidated the molecular mechanisms inherent in the immune response against potential pathogens. Hence, it became clear that both AMPs and some stress management genes encode key proteins involved in the insect’s response to pathogens [30,32]. Among the best characterized ones are those responsible for the regulation of cytokines (*C7 Contig 15362*), those linked to the phagocytosis and related to protein binding (*C8 Contig 19101*) and those involved in inflammatory responses (*C3 Contig 15265*, *C4 Contig 290595*, *C5 Contig 21310*, *C6 Contig 1327*) [30,32,33].

Data regarding the cellular and humoral insect defenses during the infectious process can provide new information on the host–pathogen interaction. Furthermore, elucidating the mechanisms of *S. brasiliensis* infection in the invertebrate model may contribute to the future detection of new therapeutic targets, as well as to the description of promising molecules for controlling this mycosis. Thus, the objective of the present study was to monitor *G. mellonella’s* phenotypic and genotypic responses against *S. brasiliensis*. To the best of our knowledge, the present study is the first to evaluate the defense-related gene expression of *G. mellonella* in response to infection with a dimorphic fungus.

## 2. Materials and Methods

### 2.1. Strains and Culture Conditions

The reference strain of *Sporothrix brasiliensis* used in this work was the American Type Culture Collection isolate, ATCC MYA 4823. The yeast cells were maintained under cryopreservation at −80 °C in the mycotheque of the Center for Microorganisms’ Investigation (CIM-UFF) until their reactivation for the experiments. For this purpose, the cryotubes were defrosted with subsequent replicas in a Yeast Extract Peptone Dextrose (YPD) medium, with incubation under shaking at 37 °C for 5 days. The microbial growth in the broth was then centrifuged at 2000× *g* for 5 min, washed with sterile Phosphate Buffered Saline (PBS; Fujifilm Irvine Scientific, Santa Ana, CA, USA) and suspended in the same buffer. After these processes, standardized suspensions of 1 × 10^7^ yeast/larva were prepared using a hemocytometer [20,21].

### 2.2. Galleria mellonella Survival Assays

The invertebrate biomodel investigated was kindly provided from a well-established colony by the Biofilms and Microbial Diversity Laboratory of the Federal University of Rio Grande do Sul. The survival curves were performed with larvae in the final larval stage of development, weighing between 0.2 g and 0.3 g, with uniform coloration, without spots or signs of melanization [34]. The inoculation of the fungus was subcutaneous, with 10 μL of the inoculum applied in PBS with a Hamilton syringe (701N, Caliber 26, Hamilton Company, Reno, NV, USA) in the last left proleg, as recommended [21,34]. The infected larvae were grouped by experimental condition, kept at 37 °C in 14 cm Petri dishes and monitored daily for survival. Lack of movement and extensive melanization of the body were both taken as indicators of animal death. As a control, two groups of animals were selected—one group with only the physical injury of the needle (naive) and the other injected with PBS—in order to assess mortality from animal handling and mechanical injury. Each group, including the control, consisted of 30 larvae. These experiments were conducted in triplicate.

### 2.3. Determination of Fungal Load during the Survival Curve

To monitor the fungal load during the different phases of infection, an experiment with ten (10) inoculated larvae was conducted with the same inoculum (1 × 10^7^ yeast/larva) and under the same temperature conditions, in triplicate, as detailed earlier in the survival curve. One larva was randomly selected from the pool daily, previously cleaned with ethanol 70%, and then, the hemolymph was collected. For this purpose, the larvae were sectioned with a sterile scalpel on the lower part, and 30 μL of hemolymph was collected from each individual. Then, the hemolymph was immediately placed in an Eppendorf^®^ tube containing physiological saline buffer for insects in a 1:10 proportion (IPS: 150 mM sodium chloride, 5 mM potassium chloride, 10 mM Tris-HCl, pH 6.9, 10 mM EDTA and 30 mM sodium citrate) [35]. Soon after, serial dilutions were performed on Sabouraud agar plates plus Chloramphenicol (Sigma-Aldrich, St. Louis, MO, USA) incubated for seven days at 25 °C. After this period, one of the colonies was chosen for observation of confirmatory micromorphology of the genus *Sporothrix* using methyl blue staining. Once the genus was detected, all colony-forming units (CFUs) were counted as described by Clavijo-Giraldo and colleagues [21].

### 2.4. Determination of Hemocyte Density in Hemolymph

Similar to the methodology used for hemolymph collection described in the previous paragraph, hemocyte density was estimated from 30 μL of hemolymph diluted in IPS in a 1:10 proportion. The number of hemocytes was counted using a hemocytometer [36]. The experiment was performed in triplicate, and the results were expressed in hemocytes/mL. In parallel to the quantitative analysis, qualitative notes of phenomena such as nodule formation, as well as yeast adhered to the hemocytes, were made.

### 2.5. Analysis of Gene Expression

#### 2.5.1. RNA Extraction and cDNA Synthesis

For the RNA extraction procedure, 20 control larvae with only the physical injury and 20 larvae inoculated with 1 × 10^7^ *S. brasiliensis*/larva were selected. The assays investigating the dynamics of gene expression were performed at three time points of the survival curve: on days one, five and eight (D1, D5 and D8) after infection with *S. brasiliensis*. For each incubation time (D), six (06) larvae from each group were randomly selected, cooled on dry ice and conditioned in microtubes for total RNA extraction from the tissue. For this purpose, the larvae were macerated using a sterile pestle, and the extraction was performed with SV Total RNA Isolation System kit (Promega Corporation, Madison, WI, USA), following the manufacturer’s recommendations. The total RNA was transcribed into complementary DNA (cDNA) using GoScript Reverse Transcription System Kit (Promega Corporation, Madison, WI, USA), according to the manufacturer’s instructions. In the cDNA synthesis, in addition to the reverse transcriptase, the DNase enzyme was also used to eliminate possible contaminating DNA in the sample. The cDNA was then quantified in the Quantus Fluorometer (Promega Corporation, Madison, WI, USA) using the QuantiFluor One dsDNA System kit (Promega Corporation, Madison, WI, USA). Finally, samples were diluted to the final use concentration of 2.5 ng/μL. All these experiments were performed in triplicate.

#### 2.5.2. Reverse Transcription–Quantitative Polymerase Chain Reaction (RT-qPCR)

The quantification of gene expression was performed by RT-qPCR using the Power SYBR™ Green PCR Master Mix (Thermo Fisher Scientific, Carlsbad, CA, USA). The gene expression levels related to the production of Gallerimycin and Galiomycin AMPs, in addition to *C7 Contig 15362* and *C8 Contig 19101* genes, were evaluated at the three time points of infection. Gene *β-actin* was used as a normalizing reference control to monitor the amount of housekeeping gene not involved in the immune response, using the same cDNA preparations. The primer sequences used are specified in Table 1. The group of non-inoculated larvae was used as a control, which was compared with the group inoculated with the fungal pathogen. The samples and controls were tested in triplicate, and all reactions were performed in 96-well plates with a final volume of 15 μL, consisting of 7.5 μL of Power SYBR™ Green PCR Master Mix, 300 nM of each primer, 7.5 ng of cDNA and DEPEC water (InvitrogenTM, Carlsbad, CA, USA) of the total volume.

The reaction was performed in the 7500 Fast Real-Time PCR System (Applied Biosystems, Framingham, MA, USA) with the following conditions: initial heating at 50 °C for 2 min, denaturation at 95 °C for 2 min, followed by 40 cycles of denaturation at 95 °C for 15 s, annealing and extension at temperatures ranging from 55 to 60 °C for 1 min, depending on each primer (Table 1). In the end, a melting curve was performed to verify the specificity of the reaction: 95 °C for 15 s, 60° for 1 min, followed by 30 s at 95 °C. The means of the threshold cycle (cT) values, measured in triplicate, were used to calculate the expression of the target genes. The results were obtained as relative gene expression values (based on the formula: 2^−ΔΔCT^) compared to the reference gene expression—*β-actin*—resulting in a value equal to 1.

### 2.6. Statistical Analyses

The survival analyses of *G. mellonella* infected with fungal pathogens were estimated using the Kaplan–Meier survival curve, in which the log-rank test was used to compare the groups in terms of survival. The evolution of fungal load over days and the relationship between the hemocyte count and CFU were analyzed using simple linear regression models. Adherence to the normal distribution of the variables was established using the Shapiro–Wilk test. The Kruskal–Wallis test and Dunn’s post hoc test were used to check the dynamics of gene expression as a function of day for each experimental group. The Mann–Whitney test was used to compare the relative quantification of genes on days D1, D5 and D8 as a function of the non-inoculated and inoculated groups. All experiments (survival curves, including larvae—individual and groups—hemocyte counts, fungal loads) were performed in triplicate. The significance level adopted was 5%, and the software used was R, version 4.1.2.

## 3. Results

### 3.1. Galleria mellonella Survival Assays

**Figure 1:**
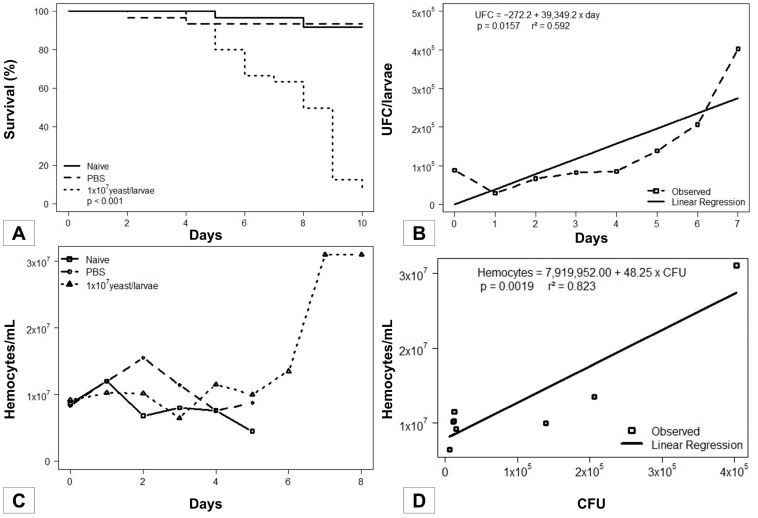
Phenotypic investigation of the infection dynamics in the invertebrate host *Galleria mellonella* against the fungal pathogen *Sporothix brasiliensis.* (**A**) Survival curve performed up to the tenth day of infection. (**B**) Monitoring of the fungal load from the insect’s hemolymph during the infection process. (**C**) Hemocyte count performed among insects in the control groups and the group infected with *S. brasiliensis.* (**D**) Parallel drawn between insect cellular immune response and fungal load along the survival curve.

The survival curve showed that larvae inoculated with 1 × 10^7^ yeast of *S. brasiliensis* started dying on day 4, while the majority of all naive larvae and those inoculated with PBS only remained unaltered (*p* < 0.001; Kaplan–Meier), reaching the pupal stage in the last days of the curve. The results are presented in Figure 1A.

### 3.2. Fungal Load during the Infection

Parallel to the survival curve, daily collection followed by hemolymph serial dilutions and subsequent plating were performed to obtain the CFUs. Colonies whose macromorphology was compatible with the genus *Sporothrix* were further evaluated using micromorphological analyses, evidencing thin, hyaline, septate, branched hyphae and conidia arranged in flower-like structures. Figure 1B shows the evolution of the fungal load along the survival curve, with increasing values from 6 × 10^3^ up to 4 × 10^5^ CFU/larva ($\bar{x}$ = 1.01 × 10^5^ s.d. ± 1.4 × 10^5^). A direct correlation of the increase in *S. brasiliensis* yeasts over the days was observed (*p* = 0.0157).

### 3.3. Sporothrix brasiliensis–Galleria mellonella Interaction

Daily hemocyte counting allowed the detection of increasing insect cellular defense generated by the fungus, verified by values from 6.4 × 10^6^ up to 3.0 × 10^7^ hemocytes/mL along the curve ($\bar{x}$ = 1.28 × 10^7^; s.d. ± 7.6 × 10^6^). The hemocyte count of the larvae from control groups (naive and PBS) was performed until D5, since afterward, both began to develop into pupae. In contrast, larvae infected with *S. brasiliensis* showed slower development in the applied experimental conditions (Figure 1C).

The hemocyte count associated with the determination of the fungal load along the survival curve allowed the evaluation of the *Sporothrix brasiliensis–Galleria mellonella* interaction dynamics. The progression of infection through the fungal pathogen triggered significant, proportional growth in insect hemocyte density (*p* = 0.0019; r^2^ = 0.823; Figure 1D).

### 3.4. Gene Expression

#### 3.4.1. Antimicrobial Peptides

**Figure 2:**
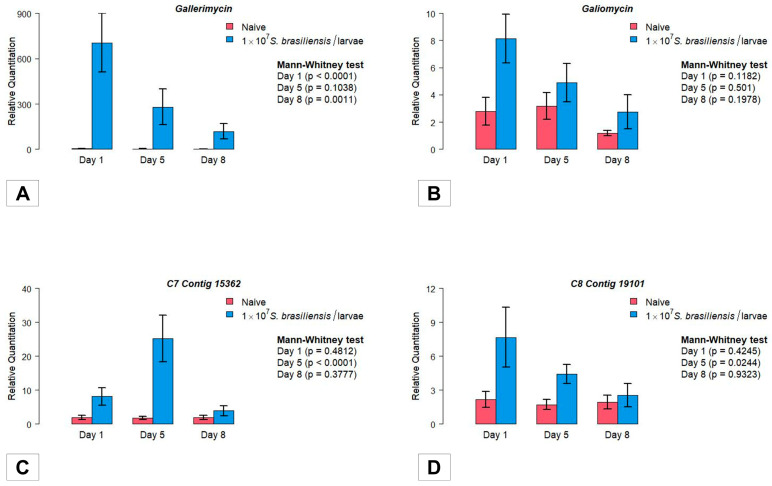
Investigation of the gene expression profile of the invertebrate host *Galleria mellonella* following infection with the pathogenic fungus *Sporothix brasiliensis*. Relative quantification of genes encoding antimicrobial peptides (**A**) Gallerimycin, (**B**) Galliomycin. Additionally, the stress manager genes (**C**) *C7 Contig 15362* and (**D**) *C8 Contig 19101*. The units on the Y-axis were calculated based on the 2^−ΔΔCT^ method and are expressed as mean and standard error of the mean (s.e.m.). Each gene was normalized and compared to the expression of control (naive) insects using the *β-actin* reference gene. The Mann–Whitney test was used to compare the relative quantification of genes, and a *p* ≤ 0.05 value was considered significant.

The gene encoding the AMP Gallerimycin showed higher early expression from the first day after infection (D1; Figure 2A) compared to naive larvae (*p* < 0.0001; Mann–Whitney test). The higher expression in *S. brasiliensis* infected larvae remained during the subsequent days D5 (*p* > 0.05) and D8 (*p* = 0.0011; Mann–Whitney test). In parallel, comparing the intragroup *Gallerimycin* gene expression, a significant reduction was observed in the infected larvae (*p* = 0.0055; Kruskal–Wallis test), contrary to the expression observed in the control group (*p* = 0.9968; Figure S1A; Supplementary Materials). 

The expression of the gene encoding Galiomycin showed a trend similar to Gallerimycin, but the difference compared to the controls did not rise to a level of statistical significance among the two experimental groups, as well as following the intragroup analysis (*p* > 0.05; Mann–Whitney test; Figure 2B and Figure S1B, Supplementary Materials).

#### 3.4.2. Stress Managing Genes

The *C7 Contig 15362* mRNA expression analysis differs among the investigated groups at D5 post-inoculation (*p* < 0.0001; Mann–Whitney test). After this period, the expression decreases and exhibits its lowest values at D8 (Figure 2C). Analysis of the intragroup gene expression showed a significant difference among the three evaluated time periods (*p* = 0.0024; Kruskal–Wallis test; Figure S1C), with higher levels of mRNA production at D5. This difference was not observed in non-infected larvae among the evaluated days (*p* > 0.05; Supplementary Materials).

The expression of *C8 Contig 19101* showed an initial, non-significant increase in mRNA expression from the first day following *S. brasiliensis* inoculation (D1; *p* > 0.05). However, at D5, an important increase in *C8 Contig 19101* gene expression was detected compared to naive larvae (Figure 2D). In the intragroup comparison, *C8 Contig 19101* mRNA expression did not vary significantly among D1 × D5 × D8 (*p* > 0.05; Kruskal–Wallis test; Figure S1D).

## 4. Discussion

Although sporotrichosis is a disease of great importance to public health, numerous aspects of the pathogen–host relationship are yet to be elucidated, mainly for the main host of this zoonosis: the domestic feline [1,39]. As a matter of fact, such investigation requires an appropriate experimental model. For decades, the murine model has been used as the in vivo gold standard model for pathogenicity mechanisms of distinct microorganisms [40], including *Sporothrix* spp. [41]. However, in recent times, the scientific community has been expressing ethical and social concerns towards the rationalization of animal model use [29,42,43].

Therefore, aiming at obtaining an alternative approach to mammalian models, several invertebrate models of infection have been studied, with emphasis on *Galleria mellonella* [44,45]. In spite of the previous use of this insect for the investigation of different fungal and bacterial infections [19,46], studies concerning the *Sporothrix* genus pathogenicity are still limited. Actually, only four previous studies investigated the *S. brasiliensis–G. mellonella* binomial, based on the description of phenotypic aspects [20,21,22,23]. Therefore, this is the first study to draw a parallel between *G. mellonella* innate-immunity-related gene expression and deleterious events during *Sporothrix* infection.

The results obtained in the survival curve of *S. brasiliensis* infection in *G. mellonella* standardization showed that the ideal yeast inoculum for observing mortality and infection dynamics was 1 × 10^7^ yeast/larva, since lower fungal loads (1 × 10^5^ and 1 × 10^6^ yeast/larva) did not allow such observations. Furthermore, the best temperature condition was 37 °C. These results were partially similar to those described by Clavijo-Giraldo and colleagues [21]. For more efficient killing with the fungal pathogen, these authors concluded that temperatures closer to the natural mammalian host are required to maintain all virulence attributes expressed by the yeast morphology. However, a less concentrated inoculum of 1 × 10^5^ yeast/larva was sufficient for these authors to obtain an appropriate survival curve. It is believed that this difference may be related mainly to the lineage of the larva used or even uncontrolled environmental conditions. On the other hand, Freitas and co-authors [20] used the same fungal load of 1 × 10^7^ yeast/larva, since when testing lower concentrations (1 × 10^4^ yeast/larva and 1 × 10^6^ yeast/larva), they did not observe mortality in the survival curve. In addition, the temperature of 37 °C was also recommended for the experiment, corroborating the results obtained here in both aspects.

Daily monitoring of *S. brasiliensis* load during the infection curve provided progressively increasing values, ranging from 1.2 × 10^4^ to 4 × 10^5^ CFU per animal. An average fungal load ranging from 1.8 × 10^5^ to 2.4 × 10^5^ CFU per animal was previously described [22] after 24 h of infection. Gandra and colleagues [31] observed that by inoculating 1 × 10^7^ *Candida albicans*/larva, the CFUs ranged from 1.0 × 10^8^ to 1.0 × 10^12^ at 6, 24 and 48 h. These data reinforce the suitability of *G. mellonella* as a model of yeast infection, since it differentially mirrors the *Candida* and *Sporothrix* fungal load increases, such as those previously described for mammalian hosts [47,48].

It is noteworthy to mention the proportional increase in hemocytes in response to *S. brasiliensis’s* higher fungal loads, beginning on the fifth day of the curve. As a matter of fact, on the fifth day, the major phenotypic event registered was the beginning of larva death. Thus, it is possible to hypothesize that larva morbidity is a result of higher fungal loads as the immune system—although proportionally stimulated, reaching greater hemocyte recruitment—fails [26,49].

Lozoya-Pérez and collaborators [22] described a similar *S. brasiliensis*–*G. mellonella* approach at a single specific point of the survival curve. The CFU and hemocyte counts from the insect’s hemolymph were obtained 24 h after yeast inoculation, previously grown in distinct culture media. CFU values of between 1.8 × 10^5^ and 2.4 × 10^5^ and hemocyte values of between 3.9 and 9.2 × 10^6^ hemocytes/mL were found, depending on the culture medium used. Such values were lower than those found in the present study (6.4 × 10^6^ to 3.0 × 10^7^ hemocytes/mL). This difference may be related to the 100x higher inoculum of *S. brasiliensis* yeast used in the present study protocol.

In spite of the increasing amount of literature involving the use of *G. mellonella* as an invertebrate biomodel, little is known about its defense strategies against *S. brasiliensis*. Even though the present study was able to establish a comparison with the previous work concerning the phenotypic events of the *Sporothrix*–*G. mellonella* interaction [21,22,50], we are limited to few previous publications describing the molecular aspects of the *S. schenckii* infection [50]. In fact, the gene expression data for such experimental scenario are limited to the genus *Candida* [31,44].

To the best of our knowledge, this is the first study investigating *G. mellonella* phenotypic and molecular events triggered through infection with the most virulent *Sporothrix* species, *S. brasiliensis*. In contrast, a considerable number of published works are available on the *G. mellonella* humoral response to the *Candida* species. These works quantify the expression of AMPs promoter genes with distinct purposes—among them, the description of the larval immune response profiles under different infection protocols and the evaluation of potential antimicrobial drugs. By respecting the differences inherent to each fungal species, these findings were used as parameters in order to discuss the findings of the present study.

Given the previous works highlighting the importance of the *G. mellonella* AMPs encoding genes *Gallerimycin* and *Galiomycin*, during both filamentous and yeast fungal infections [29,31], this study sought to quantify these gene expressions after infection with the dimorphic fungus *Sporothrix brasiliensis*. While *Gallerimycin* presented an expressive increase in gene expression along the survival curve, *Galiomycin* remained unaltered. These results suggest that the AMP Gallerimycin plays a relevant role in the insect’s response to *S. brasiliensis* infection, as previously described during *Candida* spp. infection [31,51]. Contrarily, Dekkerová-Chupáčová and co-authors [12] observed that the inoculation of 2 × 10^5^ yeast/larva of *C. albicans* and *C. dubliniensis* in *G. mellonella* triggered an expressive increase in AMP coding genes, especially *Galiomycin*.

The differential relevance of the *G. mellonella Galiomycin* gene expression during *Candida albicans* and *dubliniensis* versus *S. brasiliensis* infections may be explained by the fact that the first is a well-known microbiota yeast member, interacting with vertebrates over millions of years of evolution [52], while the *Sporothrix* species evolved from saprophytic mycelial fungi recently adapted to parasitism via thermodimorphism [53]. Likewise, differences in virulence and cell wall composition among these fungi could also be involved. From this perspective, *Sporothrix* species may be in the process of adapting gene expression in response to animal organic matter [2,53].

In addition to positive regulation, time-dependent regulation was observed, and *Gallerimycin* evidenced a significant increase on days D1 and D8. Such result partially corroborates that obtained by Dekkerová-Chupáčová and co-authors [12]. They noted that the maximum upregulation of both genes was shown at 24 h post-infection, but as early as 1 h into the infection, a positive regulation of expression was already observed. More specifically, the gene encoding *Gallerimycin* was up to 1.3-fold higher (1 h post-infection) and 3.2-fold higher (24 h post-infection) in larvae infected with *C. albicans* compared to *C. dubliniensis.* Meanwhile, for *Galiomycin,* we observed up to 3.7-fold (1 h post-infection) and 7.1-fold (24 h post-infection) relative upregulation in *C. albicans*-infected larvae. The upregulation of both genes decreased at 48 h post-infection with these two species.

In this regard, the difference in gene expression analysis intervals was based on the metabolic characteristics of the fungal species. *Candida* spp. demonstrated considerably faster growth both In Vitro and in vivo, in the invertebrate model itself, compared to *Sporothrix* spp. [13]. Moreover, given the scarcity of information regarding the gene expression of *G. mellonella* against *Sporothrix* spp., the choice of day (D) for gene expression analysis was defined according to the defense phenomena of the larvae observed in response to the fungus, such as melanization, mobility changes and the onset of mortality.

Concerning the results observed in the expression of stress management genes, *C7 Contig 15362* and *C8 Contig 19101* showed a significant difference in gene expression among the experimental groups on the fifth day (D5) of infection. Drawing a parallel with the study conducted by Melo and collaborators [30] evaluating *G. mellonella* gene expression after *C. albicans* infection versus different antifungal therapies at 24 and 48 h, it was also possible to observe a positive regulation of both genes in the infected, untreated larvae. A relevant point is that in this case, the *C8 Contig 19101* gene was about four times overexpressed compared to the C7 *Contig 15362* gene.

In the present study, it was possible to observe that the expression of the genes studied is not only time dependent but also alternately modulated, since, when comparing the genes responsible for encoding AMPs with those of stress management, this significant increase D-day varied. *C7 Contig 15362* and *C8 Contig 19101* are considered stress management genes and showed a significant increase in expression on day 5 post-infection. Interestingly, this moment coincided with the onset of mortality and deleterious phenotypic changes observed during the survival curve, as well as with the beginning of the expressive increase in defense cells and fungal load. In contrast, genes encoding AMPs, especially *Gallerimycin*, showed an immediate response verified by the intense mRNA encoding as early as the first post-infection day. This finding is compatible with the participation of the AMPs, previously characterized as an early element of humoral immunity against the infectious process [54,55]. For the same reason, different authors determine the evaluation protocols in hours, both in the investigation of the infectious process with *Candida* spp. [12,31,51] and with *Aspergillus niger* [17].

This work is the first to evaluate the gene expression related to the defense of the insect *G. mellonella* in response to infection with a dimorphic fungus. Most authors have dedicated themselves to the investigation of yeasts and, among these, of the genus *Candida* [12,30,31]. Among the filamentous fungi, previous investigations considered *Aspergillus niger* [17] and *Fusarium oxysporum* [56].

The possibility that filamentation occurred during the infectious process in *G. mellonella* cannot be excluded. Thus, the comparative discussion of some of the parameters evaluated here is limited. It is important to add the fact that the presence of hyphae of *S. brasiliensis* has been reported In Vitro after feline phagocyte exposure [57] and either during human or animal parasitism (manuscript in preparation). It is known that the fungal cell wall undergoes profound transformation, altering—during the dimorphic transition—β-1-3-glucans to α-glucans, posing an immediate challenge to the host immune response (PAMPs) [17,58]. However, only studies considering the distinct periods within the survival curve and also the evaluation of potential changes in fungal cell morphology will be able to answer whether the verified gene expression was exclusively yeast-triggered and/or yeast-targeted.

Another acknowledged limitation is the absence of data regarding gene expression and protein translation or its antimicrobial activity. However, because this study represents a pioneering investigation in the area, new perspectives and methodologies can be generated and improved in order to overcome the scarcity of data in the literature and elucidate the aspects of this relevant pathogen–host interaction. The present work opens new doors of investigation on the pathogenesis with *Sporothrix* spp., as well as offering a better characterized model for the research of potential antifungal drugs effective for the control of this important zoonosis.

## Figures and Tables

**Table 1:** Genes quantified during the fungal infection process of *Galleria mellonella* with *Sporothrix brasiliensis*. The references used, as well as the primer sequences.

Gene	NCBI Genbank References	Sequence (5′–3′)	Annealing Temperature
*Galiomycin*	AY528421.1	F-TCCAGTCCGTTTTGTTGTTG	60 °C
	[37]	R-CAGAGGTGTAATTCGTCGCA	
*Gallerimycin*	AF453824.1	F-GAAGATCGCTTTCATAGTCGC	60 °C
	[37]	R-TACTCCTGCAGTTAGCAATGC	
*C7 Contig 15362*	*Contig 15362*	F-CGAGCTAAAGACAGGCGATT	58 °C
	[30]	R-TCACCTGCGGTTGAATCATA	
*C8 Contig 19101*	*Contig 19101*	F-ATTGCTAGCCAGGTTCAGGA	60 °C
	[30]	R-AGCTATTTGGCGGAAACTCA	
*β-actin*	[38]	F-GGACTTGTACGCCAACACAG R-CCACATCTGCTGGAATGTCG	55 °C

## Data Availability

The data presented in this study are available within the article.

## Supplementary Materials

The following supporting information can be downloaded at: https://www.mdpi.com/article/10.3390/jof9111053/s1. Figure S1: Investigation of the gene expression profile of the invertebrate host *Galleria mellonella* in infection by the pathogenic fungus *Sporothix brasiliensis*. Dynamics of gene expression observed according to day, for each experimental group. (A) *Gallerimycin*, (B) *Galliomycin* and the stress manager genes (C) *C7 Contig 15362* and (D) *C8 Contig 19101*. The units on the Y axis were calculated based on the 2^−ΔΔCT^ method, and are expressed as mean. Each gene was normalized and compared to the expression of control (naive) insects using the *β-actin* reference gene. The Mann-Whitney test was used to compare the relative quantification of genes and a *p* ≤0.05 value was considered significant.
