# Supplementary material for: Sporothrix brasiliensis Infection Modulates Antimicrobial Peptides and Stress Management Gene Expression in the Invertebrate Biomodel Galleria mellonella
Source: J Fungi (Basel). 2023 Oct 27;9(11):1053. doi: 10.3390/jof9111053 (PMC10672515; doi:10.3390/jof9111053)
Supplement: Supplementary file 1 [file jof-09-01053-s001.zip › jof-2612413-supplementary.pdf]

Supplementary material

Sporothrix brasiliensis Infection Modulates Antimicrobial Peptides and Stress Management Gene Expression in the Invertebrate Biomodel *Galleria mellonella*

Nathália Faria Reis [1], Myrela Conceição Santos de Jesus [1], Lais Cavalcanti dos Santos Velasco de Souza [1], Lucas Martins Alcântara [1], Julia Andrade de Castro Rodrigues [1], Simone Cristina Pereira Brito [1], Patrícia de Azambuja Penna [2], Cecília Stahl Vieira [2], José Rodrigo Santos Silva [3], Bruno de Araújo Penna [4], Ricardo Luiz Dantas Machado [1], Hector M. Mora-Montes [5] and Andréa Regina de Souza Baptista [1,6,*]

[1] Center for Microorganisms' Investigation, Department of Microbiology and Parasitology, Biomedical Institute, Federal Fluminense University, Niterói 24020-141, RJ, Brazil; nathaliafariareis@id.uff.br (N.F.R.); myrelaj@id.uff.br (M.C.S.d.J.); laiscavalcanti@id.uff.br (L.C.d.S.V.d.S.); martins_lucas@id.uff.br (L.M.A.); julia_andrade@id.uff.br (J.A.d.C.R.); scpbrito@id.uff.br (S.C.P.B.); ricardomachado@id.uff.br (R.L.D.M.)

[2] Laboratory of Insect Biology, Federal Fluminense University, Niterói 24220-900, RJ, Brazil; patriciaazambuja@id.uff.br (P.d.A.P.); ceciliastahl@gmail.com (C.S.V.)

[3] Department of Statistics and Actuarial Sciences, Federal University of Sergipe, São Cristóvão 49107-230, SE, Brazil; rodrigo.ufs@gmail.com

[4] Laboratory of Gram-Positive Cocci, Biomedical Institute, Fluminense Federal University, Niterói 24020-141, RJ, Brazil; bpenna@id.uff.br

[5] Department of Biology, Division of Natural and Exact Sciences, Campus Guanajuato, University of Guanajuato, Guanajuato 36050, Mexico; hmora@ugto.mx

[6] Rede Micologia RJ—Fundação de Amparo à Pesquisa do Estado do Rio de Janeiro (FAPERJ), Rio de Janeiro 21941-630, RJ, Brazil

* Correspondence: andrearegina@id.uff.br

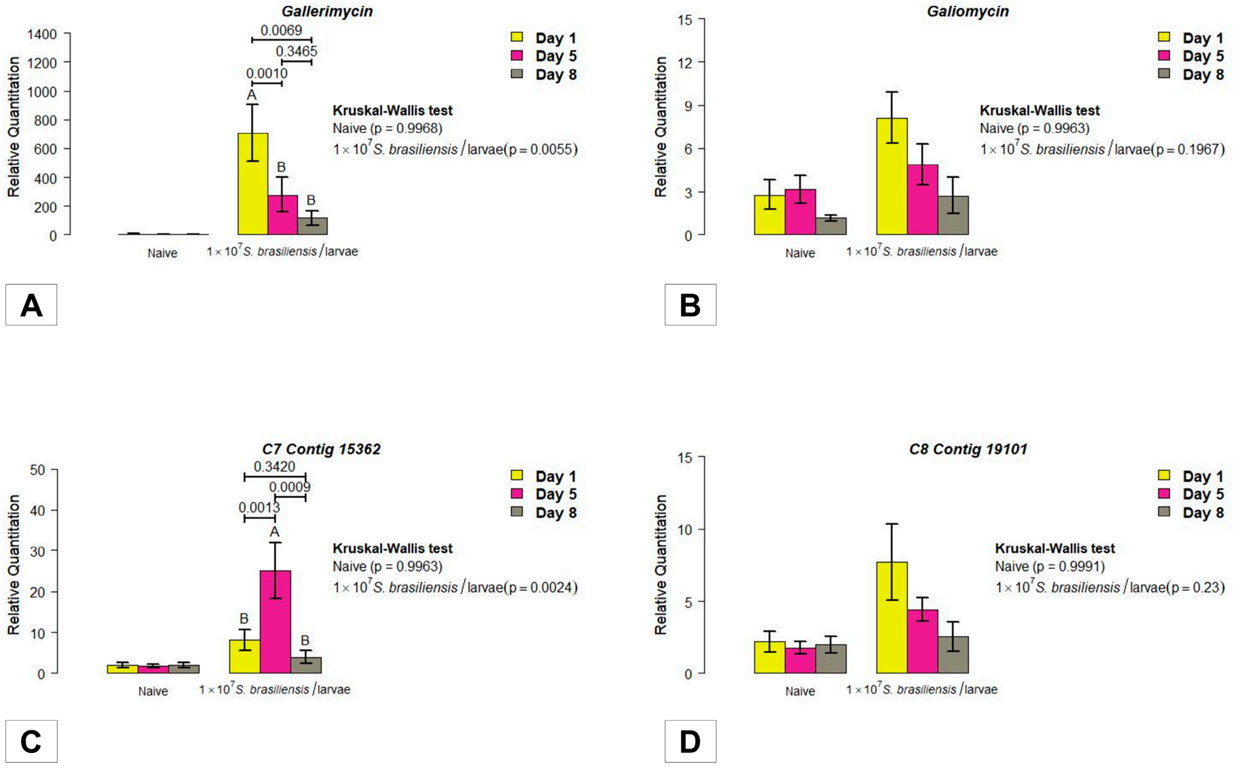

Figure S1: Investigation of the gene expression profile of the invertebrate host *Galleria mellonella* in infection by the pathogenic fungus *Sporothix brasiliensis*. Dynamics of gene expression observed according to day, for each experimental group. **(A)** *Gallerimycin*, **(B)** *Galliomycin* and the stress manager genes **(C)** *C7 Contig 15362* and **(D)** *C8 Contig 19101*. The units on the Y axis were calculated based on the $2^{-\Delta\Delta CT}$ method, and are expressed as mean. Each gene was normalized and compared to the expression of control (naive) insects using the *β-actin* reference gene. The Mann-Whitney test was used to compare the relative quantification of genes and a *p* ≤0.05 value was considered significant.
